# Toxicity and utilization of chemical weapons: does toxicity and venom utilization contribute to the formation of species communities?

**DOI:** 10.1002/ece3.1595

**Published:** 2015-07-14

**Authors:** Fabian L Westermann, Iain S McPherson, Tappey H Jones, Lesley Milicich, Philip J Lester

**Affiliations:** 1 Centre for Biodiversity and Restoration Ecology, Victoria University of WellingtonPO Box 600, Wellington, New Zealand; 2 Department of Chemistry, Virginia Military InstituteLexington, Virginia, 24401

**Keywords:** Ants, competition, invasive species, *Linepithema humile*, *Monomorium*, toxicity, venom

## Abstract

Toxicity and the utilization of venom are essential features in the ecology of many animal species and have been hypothesized to be important factors contributing to the assembly of communities through competitive interactions. Ants of the genus *Monomorium* utilize a variety of venom compositions, which have been reported to give them a competitive advantage. Here, we investigate two pairs of *Monomorium* species, which differ in the structural compositions of their venom and their co-occurrence patterns with the invasive Argentine ant. We looked at the effects of *Monomorium* venom toxicity, venom utilization, and aggressive physical interactions on *Monomorium* and Argentine ant survival rates during arena trials. The venom toxicity of the two species co-occurring with the invasive Argentine ants was found to be significantly higher than the toxicity of the two species which do not. There was no correlation between venom toxicity and *Monomorium* survival; however, three of the four *Monomorium* species displayed significant variability in their venom usage which was associated with the number of Argentine ant workers encountered during trials. Average *Monomorium* mortality varied significantly between species, and in *Monomorium smithii* and *Monomorium antipodum,* aggressive interactions with Argentine ants had a significant negative effect on their mortality. Our study demonstrates that different factors and strategies can contribute to the ability of a species to withstand the pressure of a dominant invader at high abundance, and venom chemistry appears to be only one of several strategies utilized.

## Introduction

Venom toxicity and utilization mode are two important features in the ecology of many animal species. A wide range of organisms produce and utilize toxins to hunt for prey or defend themselves and their kin from predators or competitors (Halstead and Courville 1988; Mebs 2001, 2002). Venom may also give invasive species a substantial advantage against native species due to lack of coevolutionary history (Michael Crossland and Alford 1998; Holway et al. 2002b; Albins and Hixon 2008; Greenberg et al. 2008). The means of venom deployment differ between organisms and include injection, passive application on the skin, or shooting/squirting over a distance (Mebs 2002; Warrell 2007).

Biotic resistance is an important factor limiting invaders’ impacts on a community and hindering their establishment and spread. Such biotic resistance has been found in invasive fish (Baltz and Moyle 1993), birds (Blackburn and Duncan 2001), crabs (DeRivera and Ruiz 2005), and ants (Rowles and O’Dowd 2007; Blight et al. 2010). To date only few studies have investigated closely whether venom toxicity, or its application strategy, might be an important factor in understanding why certain native species are able to successfully resist an otherwise dominant invader, while other species are displaced.

Ant venom has been associated with co-occurrence patterns in ant communities (Holway 1999) and has also been reported as means of biotic resistance against invasive species such as the Argentine ant, *Linepithema humile* (Mayr) (Sorrells et al. 2011). One study reported venom of *Monomorium rothsteini* (Forel) to be highly repellent against three *Iridomyrmex* species, and the authors concluded that venom alkaloids may be a potent yet poorly appreciated force in interference competition and possibly play an important role in the structure of ant communities (Andersen et al. 1991).

The Argentine ant is considered one of the world’s most invasive species and has established widespread colonies in many Mediterranean and subtropical regions worldwide (Suarez et al. 2001; Holway et al. 2002a; Wild 2004). It has been linked with population declines in a variety of organisms (Cole et al. 1992; Sanders et al. 2003; Rowles and O’Dowd 2009a) as well as damage to agricultural crops by supporting herbivorous pest species (Ward et al. 2010). Populations quickly reach high abundance, and estimates of 50,000–600,000 ants on a trail have been reported (Vega and Rust 2001). Such high abundances allow them to eliminate even comparatively large species (De Kock 1990). In New Zealand, the Argentine ant was first observed near Auckland and has since spread to several locations around the country (Green 1990).

One ant genus, which has repeatedly been reported to successfully withstand other competitively strong ant competitors, is *Monomorium* (Adams and Traniello 1981; Andersen and Patel 1994; Holway 1999). For example, in Australia *Monomorium* was observed to occupy baits regardless of the presence or absence of the otherwise competitively dominant “meat ant” *Iridomyrmex purpureus* (Smith), potentially because of its usage of venom alkaloids (Andersen and Patel 1994). In competitive tests between seven native ants and the Argentine ant, *Monomorium ergatogyna* (Wheeler), which was the smallest species, won 100% of the interactions (Holway 1999). Another study showed that *Monomorium minimum* (Buckley) uses chemical interference to delay competitors (Adams and Traniello 1981).

**Figure 1:**
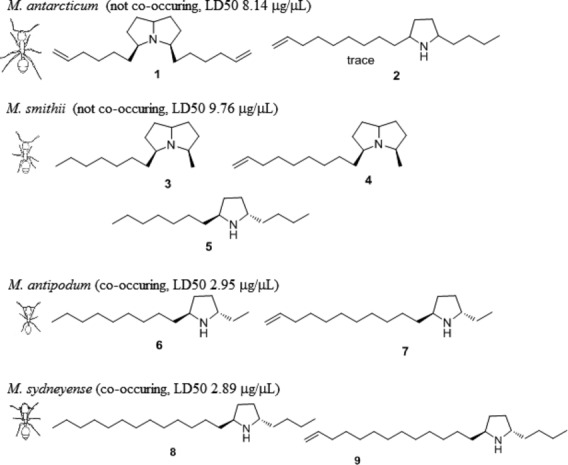
Structural formula of venom and illustrations of the four *Monomorium* species. *Monomorium antarcticum* (1) (5Z,8E)-3,5-di-(5-hexen-1-yl)-pyrrolizidine (2) and 2-butyl-5-(8-nonenyl)pyrrolidine; *Monomorium smithii* (3) (5Z,8E)-3-heptyl-5-methylpyrrolzidine, (4) (5Z,8E)-3-methyl-5-(8-nonenyl)pyrrolizidine, and (5) *trans-*2-butyl-5-heptylpyrrolidine in a 1:2:2 ratio; *Monomorium antipodum* (6) *trans*-2-ethyl-5-nonylpyrrolidine and (7) *trans-*2-ethyl-5-(10-undecenyl)pyrrolidine in a 1:1 ratio; and *Monomorium sydneyense* (8) *trans-*2-butyl-5-undecylpyrrolidine and (9) *trans-*2-butyl-5-(12-tridecenyl)pyrrolidine in a 1:2 ratio. Sketches of *Monomorium* species represent size differences of ant workers (sketches not to scale). Given LD50 values refer to the venom toxicity of the *Monomorium* venom to Argentine ant workers.

*Monomorium* ants utilize a range of venom alkaloids. Available evidence suggests that the venom chemistry of each alkaloid-producing species of *Monomorium* has a qualitatively characteristic signature (Jones et al. 2009), likely resulting in varying venom toxicity. In New Zealand, the native *Monomorium* species *M. antarcticum* (Smith) and *M. smithii* (Forel) have not been observed to co-occur with Argentine ants on larger scales, with only slight overlaps at the borders of their occupied ranges. However, the two exotic species *Monomorium antipodum* (Forel) and *Monomorium sydneyense* (Forel) have been observed to co-occur with Argentine ants in New Zealand (Lester et al. 2003) or elsewhere (Rowles and O’Dowd 2009b). The venom compositions of the native and non-native *Monomorium* species differ in one important aspect. While the venom of *M. antarcticum* and *M. smithii* contains both pyrrolizidine and pyrrolidine alkaloids, the venoms of *M. antipodum* and *M. sydneyense* contain only the pyrrolidines **6**, **7**, **8**, and **9** (Don et al. 2001). The differences in the venom chemistry between those two *Monomorium* groups may result in a general mechanism by which venom chemistry influences distribution patterns of those species and their co-occurrence with Argentine ants. Illustrations of the four study species and structural formulae of their venoms are given in Figure1.

Here, we report the results of a mechanistic study to understand the observed patterns of co-occurrence and determine the importance of chemical weapons in community patterns and biotic resistance. We performed a series of behavioral assays and venom effectiveness assessments between the invasive Argentine Ant and the four *Monomorium* species that occur in New Zealand. Our goal was to investigate whether patterns of co-occurrence previously observed in the field are related to venom toxicity and composition or its deployment method. We based our investigations on the assumption that the two *Monomorium* species (*M. antarcticum* and *M. smithii*) not co-occurring with Argentine ants may be able to exclude the Argentine ant from their habitat through biotic resistance by means of venom application as had been indicated by other studies (Adams and Traniello 1981; Andersen and Patel 1994; Holway 1999). Therefore, we hypothesized that (1) *Monomorium* species with higher venom toxicity to Argentine ants have higher survival rate when engaging Argentine ants; (2) the mortality of *Monomorium* species engaging Argentine ants should differ depending on venom utilization; and (3) in the absence of venom, aggressive interactions result in a lower survival rate of the *Monomorium* species engaging Argentine ants.

## Materials and Methods

### Ant samples

We collected nests of all four *Monomorium* species currently found in New Zealand at locations around the North Island: *M. antarcticum* (Ohariu Bay; −41.219, 174.710) *M. antipodum* (Palmerston North; −40.354, 175.606), *M. smithii* (Ohariu Bay; −41.219, 174.710), and *M. sydneyense* (Sulfur Point; −38.130, 176.265), as well as of the invasive Argentine ant *L. humile* (Petone; −41.222, 174.872). All nests were housed in nest boxes at a constant temperature of 20°C and a day/night cycle of 12/12 h. Walls of the nest boxes were covered with Fluon (DuPont™ Teflon® PTFE DISP 30), and ants were fed a diet of 20% honey water and two mealworms three times a week. Nesting tubes, consisting of a glass tube with water covered with a cotton plug and surrounded with aluminum foil, were provided.

### Arena fight and venom usage

Fighting experiments were conducted between November 2010 and June 2011. Twelve worker ants of one of the four species (*M. antarcticum*, *M. smithii*, *M. antipodum,* and *M. sydneyense*) were placed in a small container together with 20, 40, 80, or 120 Argentine ants. Six replicates were conducted for each combination of the four *Monomorium* species and the four Argentine ant densities. The mortality of *Monomorium* and Argentine ants was assessed after one, four, and 24 h. In the first 15 min, a randomly chosen *Monomorium* ant was observed and the displayed behavior in each interaction with Argentine ants scored (=individual aggression). Behavior scores were taken as follows: 0 = no interest or aggression; 1 = interest shown via antennation; 2 = ant retreats quickly; 3 = lunging, biting or leg pulling, raising of gaster, and exuding venom; and 4 = prolonged (>5 sec) incidences of aggression, individuals locked together, and fighting (Rowles and O’Dowd 2007). If prolonged fighting occurred, another ant was chosen. This was performed to achieve a maximum of twenty behavioral observations, as the observed *Monomorium* ant did not always survive the interaction. Within the first hour, all ants were also scanned at random intervals for a maximum of twenty observations, and the interaction with the highest observable aggression at this given moment was scored (= maximum aggression). This was performed for comparison, in case the interactions of a randomly chosen individual ant were not representative of the general aggression displayed within the arena (for instance if individuals selected would have been exceptionally docile compared to the others). For each observation, the type of venom usage by the *Monomorium* ants was noted either as stinging, gaster flagging/spraying, or no venom usage.

### Venom survivability

Venom survival experiments were conducted between November 2011 and February 2012. Gas chromatography–mass spectrometry (GC-MS) analyses were carried out in the EI mode using a Shimadzu QP-2010 GC-MS equipped with an RTX-5, 30 m × 0.25 mm i.d. column. The instrument was programmed from 60 to 250°C at 10°/min. The major alkaloids of the four ant species were identified by direct comparison with synthetic samples available from previous studies or prepared by the usual method of reductive amination of the appropriate 1,4-diketones, to form pyrrolidines, or 1,4,7-triketones to form pyrrolizidines (Jones et al. 1980a,b, 1988). These were (**1**) (5Z,8E)-3,5-di-(5-hexen-1-yl)-pyrrolizidine from *M. antarcticum*; (**3**) (5Z,8E)-3-heptyl-5-methylpyrrolzidine, (**4**) (5Z,8E)-3-methyl-5-(8-nonenyl)pyrrolizidine, and (**5**) *trans-*2-butyl-5-heptylpyrrolidine in a 1:2:2 ratio from *M. smithii*; (**6**) *trans*-2-ethyl-5-nonylpyrrolidine and (**7**) *trans-*2-ethyl-5-(10-undecenyl)pyrrolidine in a 1:1 ratio from *M. antipodum*; and (**8**) *trans-*2-butyl-5-undecylpyrrolidine and (**9**) *trans-*2-butyl-5-(12-tridecenyl)pyrrolidine in a 1:2 ratio from *M. sydneyense*. Alkaloid (**2**) 2-butyl-5-(8-nonenyl)pyrrolidine from *M. antarcticum* was only identified as a trace component of the venom and was therefore not included. It should also be mentioned that the venom composition of *M. smithii* differed from venom alkaloids identified in a previous study (Jones et al. 1990). The structural formulae of the venom components are given in Figure1.

We used concentrations of 1, 5, 10, 15, and 20 μg/μL of the respective venoms in ethanol, as well as a control with the pure solvent. Argentine ant workers were randomly picked from a nest box and placed in a glass vial. We anaesthetized them by aerating them with ether vapor for 10 sec and then closing the vial for 50 sec. Subsequently, twenty anaesthetized ants were then placed on filter paper in a glass Petri dish, and each ant was treated with 1 μL of venom solution. The droplet was observed to flow over the body and engulfed the ant for a moment before being soaked by the filter paper, which ensured the animals did not drown in the liquid. Ants were consequently placed into small plastic containers to awake from anesthesia. Status of the treated animals was assessed after 1 and 4 h. Ants were scored as dead if they were immobile and gentle prodding with the tip of forceps did not elicit any reaction. Ants were counted as alive if any reaction (leg or antennae movement) could be observed or when they were moving normally.

### Statistical analysis

To investigate whether *Monomorium* species with higher venom toxicity to Argentine ants have higher survival rate when engaging Argentine ants, we combined the data from our arena tests and our venom toxicity assessments. A Kaplan–Meier survival analysis was used to determine the differences in survival times in the arena fights between the four *Monomorium* species and Argentine ants by pooling the mortality rates over all densities. A probit analysis was used to assess the mortality rates of Argentine ants treated with venom of the *Monomorium* species and calculate the median lethal dosage (LD50) of the respective venoms on Argentine ants. A Linear regression analysis was then used to analyze the impact of venom toxicity on survival of the respective *Monomorium* species and the Argentine ants they encountered.

To investigate whether survival chances of *Monomorium* species engaging Argentine ants differed depending on venom utilization, we analyzed the occurrence of the three venom displays: “no venom,” “gaster flagging or spraying,” and “stinging.” We examined whether these behaviors were related to the number of Argentine ant workers encountered and to *Monomorium* survival in each trial, which was categorized as “low” (0–4 workers died), “medium” (5–8 workers died), or “high” (9–12 workers died) using Pearson chi-square tests.

To determine whether aggressive interactions resulted in a lower survival rate of the species engaging Argentine ants in arena fights, we used generalized linear models with the following setup: relative *Monomorium* mortality (number of deceased *Monomorium* workers divided by the number of *Monomorium* workers in the arena) and relative Argentine ant mortality (number of deceased Argentine ants divided by the number of Argentine ants in the arena) as dependent variable; individual aggression (displayed behavior of individual ant in each interaction with Argentine ants) and maximum aggression (interaction with the highest observable aggression at this given moment) as factors; and Argentine ant density as a covariate. The models were run as a full factorial model to investigate any interactions between the Argentine ant density and aggression. We also used a generalized linear model to look for differences in the mortality between the four *Monomorium* species, with relative *Monomorium* mortality (number of deceased *Monomorium* workers divided by the number of *Monomorium* workers in the arena) as dependent variable, *Monomorium* species as factor, and Argentine ant density as a covariate.

All data analysis was conducted using IBM SPSS Statistics v. 20.0 (IBM Corporation, Armonk, New York, United States). Figures were created using OriginPro v. 8.6. The level of significance was defined at *P* < 0.05.

The data package associated with this manuscript has been made available at the Dryad data depository (Dryad access number: doi: 10.5061/dryad.7g02r).

## Results

Our first hypothesis was that species with higher venom toxicity would have a higher rate of survival when engaging Argentine ants. The Kaplan–Meier survival analysis showed significant differences (Mantel–Cox: chi-square 336.77, df = 3, *P* < 0.001) in the survival times of the four *Monomorium* species in arena fights against Argentine ants. *Monomorium antarcticum* (mean 24 h ± 0) and *Monomorium antipodum* (mean 23.77 h ± 0.12) survived longer than *Monomorium sydneyense* (mean 21.63 h ± 0.35) and *M. smithii* (mean 20.72 h ± 0.41).

**Figure 2:**
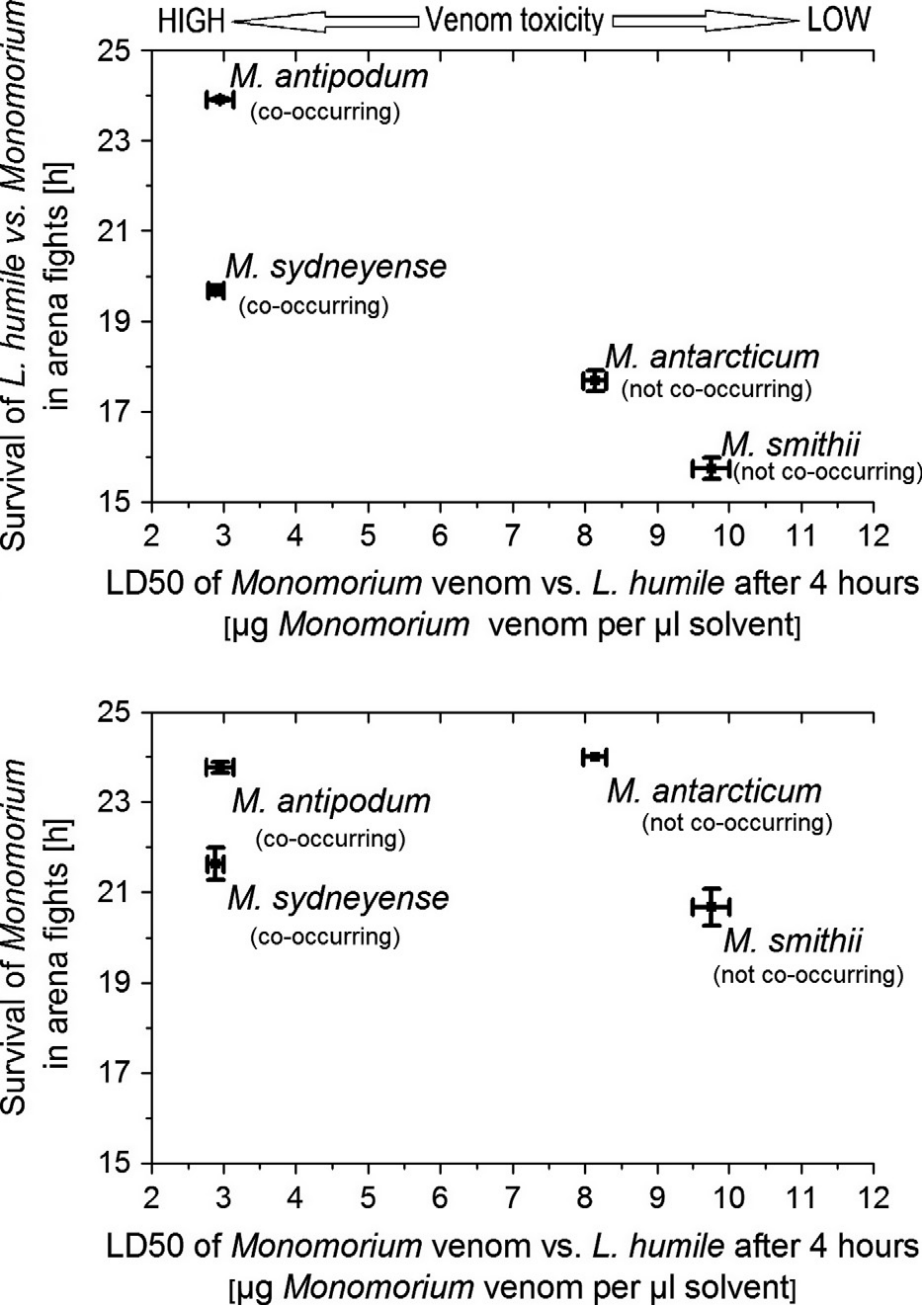
*Monomorium* toxicity and ant survival. Overall survival time of Argentine ant workers in arena fights against four *Monomorium* species correlated with the LD50 of the venom of that *Monomorium* species (top) and the overall survival time of *Monomorium* workers in arena fights against Argentine ants correlated with the LD50 of the venom of that *Monomorium* species (bottom).

The probit analysis of Argentine ant mortality rates when treated with venom of the four *Monomorium* species showed significant differences in lethality of venoms 4 h after treatment. While the venom of co-occurring *M. sydneyense* and *M. antipodum* had a relatively low LD50 (2.89 and 2.95 μg/μL), the venom of the two *Monomorium* species, which do not co-occur with Argentine ants (*M. antarcticum* and *M. smithii*), has a much higher LD50 for Argentine ant workers (8.14 and 9.76 μg/μL). We found no significant correlation between *Monomorium* survival and the toxicity of *Monomorium* venom (Pearson correlation, 1-tailed, *R* = −0.279, *P* = 0.36); however, there was a significant negative correlation between Argentine ant survival and the toxicity of *Monomorium* venom (Pearson correlation, 1-tailed, *R* = −0.954, *P* = 0.023) (Fig.2).

We hypothesized that survival chances of species engaging Argentine ants would differ depending on venom utilization. Three of the four *Monomorium* species displayed significant variability in venom usage (Table1) depending on the number of Argentine ant workers encountered (*M. antarcticum*; *N* = 444; chi-square 44.02; df = 6; *P* < 0.001; *M. smithii*; *N* = 474; chi-square 36.80; df = 6; *P* < 0.001; *M. antipodum*; *N* = 484; chi-square 32.52; df = 6; *P* < 0.001; *M. sydneyense*; *N* = 229; chi-square 2.64; df = 6; *P* = 0.853). For the species which have not been observed to co-occur with Argentine ants (*M. antarcticum* and *M. smithii*), venom usage shifted from no venom usage or gaster flagging and spraying to a high usage of stinging. For *M. antipodum*, an immediate increase in gaster flagging and spraying was observed with an increase in Argentine ant numbers.

**Table 1:** *Monomorium* venom usage depending on Argentine ant density

Species	Density	Venom			Total
		No Venom	Gaster	Stinging	
*Monomorium antarcticum* (does not co-occur; LD50 8.14 μg/μL)	20	7	24	77	108
	40	6	16	85	107
	80	0	17	91	108
	160	0	0	121	121
	Total	13	57	374	444
*Monomorium smithii* (does not co-occur; LD50 9.76 μg/μL)	20	12	46	60	118
	40	13	49	58	120
	80	7	44	67	118
	160	4	17	97	118
	Total	36	156	282	474
*Monomorium antipodum* (co-occurs; LD50 2.95 μg/μL)	20	89	12	0	101
	40	78	53	3	134
	80	82	42	0	124
	160	78	42	5	125
	Total	327	149	8	484
*Monomorium sydneyense* (co-occurs; LD50 2.89 μg/μL)	20	1	4	26	31
	40	3	12	50	65
	80	3	9	44	56
	160	1	15	61	77
	Total	8	40	181	229

Number of observed occurrences of venom utilization of four *Monomorium* species in arena trials, corresponding with the Argentine ant density encountered. Six replicates were conducted for each combination of the four *Monomorium* species and the four Argentine ant densities. The table displays the species (venom toxicity from venom survival trials included for reference), Argentine ant worker density, the sum of all displayed venom behaviors in that category (no venom, gaster flagging or spraying, and stinging), and the total number of behavior observations during trials with the corresponding density.

A significant relationship between venom utilization and mortality after 24 h was only found in *M. antipodum* (*N* = 484; chi-square 21.17; df = 4; *P* < 0.001), where physical aggression was more common in trials which resulted in low mortality, while gaster flagging was 81% higher in trials which resulted in high mortality. No significant association was found in *M. antarcticum* (*N* = 444; chi-square 5.27; df = 2; *P* = 0.07) or *M. sydneyense* (*N* = 229; chi-square 0.56; df = 4; *P* = 0.97), while *M. smithii* could not be analyzed as their mortality was always of the category “high” and therefore constant (Table2).

**Table 2:** *Monomorium* venom usage depending on *Monomorium* mortality

Species	Mortality	Venom			Total
		No venom	Gaster	Stinging	
*Monomorium antarcticum* (does not co-occur; LD50 8.14 μg/μL)	High	0	0	0	0
	Medium	0	6	71	77
	Low	13	51	303	367
	Total	13	57	374	444
*Monomorium smithii* (does not co-occur; LD50 9.76 μg/μL)	High	36	156	282	474
	Medium	0	0	0	0
	Low	0	0	0	0
	Total	36	156	282	474
*Monomorium antipodum* (co-occurs; LD50 2.95 μg/μL)	High	98	67	4	169
	Medium	46	31	0	77
	Low	183	51	4	238
	Total	327	149	8	484
*Monomorium sydneyense* (co-occurs; LD50 2.89 μg/μL)	High	5	27	122	154
	Medium	2	8	42	52
	Low	1	5	17	23
	Total	8	40	181	229

Number of observed occurrences of venom utilization of four *Monomorium* species in arena trials, corresponding with the *Monomorium* mortality rates in arena trials. The table displays the species (venom toxicity from venom survival trials included for reference), *Monomorium* mortality (low = 0–4; medium = 5–8; high = 9–12), the sum of all displayed venom behaviors in that category (no venom, gaster flagging or spraying, and stinging), and the total number of behavior observations during trials with the corresponding *Monomorium* mortality.

**Figure 3:**
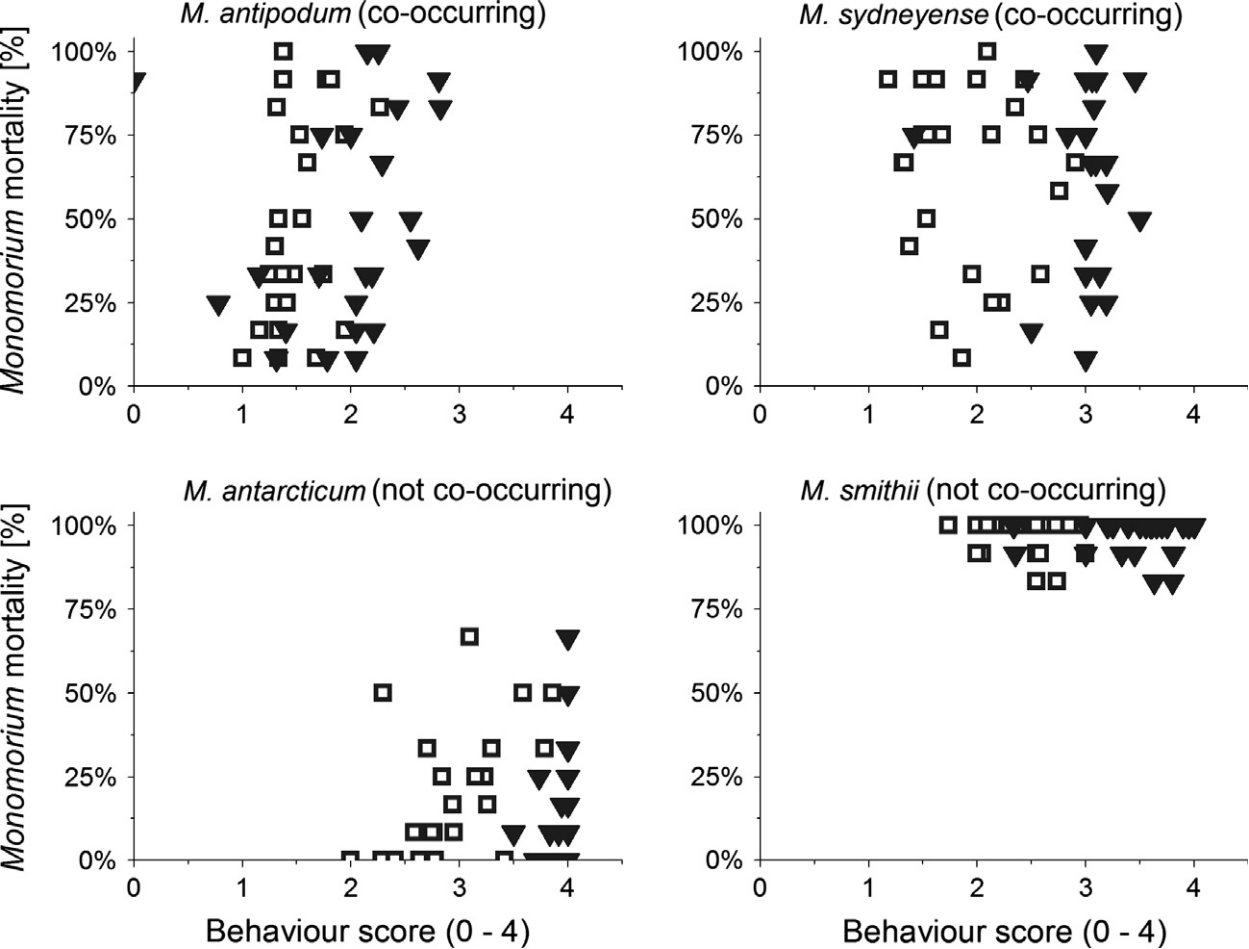
*Monomorium* mortality in arena fights. Mortality of *Monomorium* workers in arena fights against Argentine ants depending on average individual aggression within the same replicate (open squares) and average maximum aggression within the same replicate (closed triangles). Behavior scores were taken as follows: 0 = no interest or aggression; 1 = interest shown via antennation; 2 = ant retreats quickly; 3 = lunging, biting or leg pulling, raising of gaster, and exuding venom; and 4 = prolonged (>5 sec) incidences of aggression, individuals locked together, and fighting.

Our third hypothesis predicted that aggressive interactions would result in lower survival of the species engaging Argentine ants. Different patterns for mortality and behavior were observed in all four species when encountering Argentine ants (Fig.3). *Monomorium* mortality varied significantly between species (generalized linear model, *N* = 96, df = 3, Wald chi-square = 92.67, *P* < 0.001). *M. antarcticum* had the lowest (average 2%) and *M. smithii* the highest mortality (average 96.8%). Significant differences between the four *Monomorium* species were also found for the factors individual aggression (generalized linear model, *N* = 96, df = 3, Wald chi-square = 29.35, *P* < 0.001) and maximum aggression (generalized linear model, *N* = 96, df = 3, Wald chi-square = 154.23, *P* < 0.001) displayed in interactions with Argentine ants. Individual aggression had a significant effect on the mortality of *M. antipodum* (generalized linear model, *N* = 24, df = 1, Wald chi-square = 5.89, *P* = 0.015; covariate Argentine ant density df = 3, Wald chi-square = 5.85, *P* = 0.119) and *M. smithii* (generalized linear model, *N* = 24, df = 1, Wald chi-square = 9.46, *P* = 0.002; covariate Argentine ant density df = 3, Wald chi-square = 1.99, *P* = 0.574). Individual aggression had no effect on the mortality of *M. antarcticum* or *M. sydneyense*, in all cases Argentine ant density did not appear to have any significant effect. Similarly no effect of the maximum aggression on any of the *Monomorium* species survival was found, although here Argentine ant density was significant for *M. antipodum* Argentine ant density (generalized linear model, *N* = 24, df = 1, Wald chi-square = 0.66, *P* = 0.417; covariate Argentine ant density df = 3, Wald chi-square = 8.5, *P* = 0.037).

**Figure 4:**
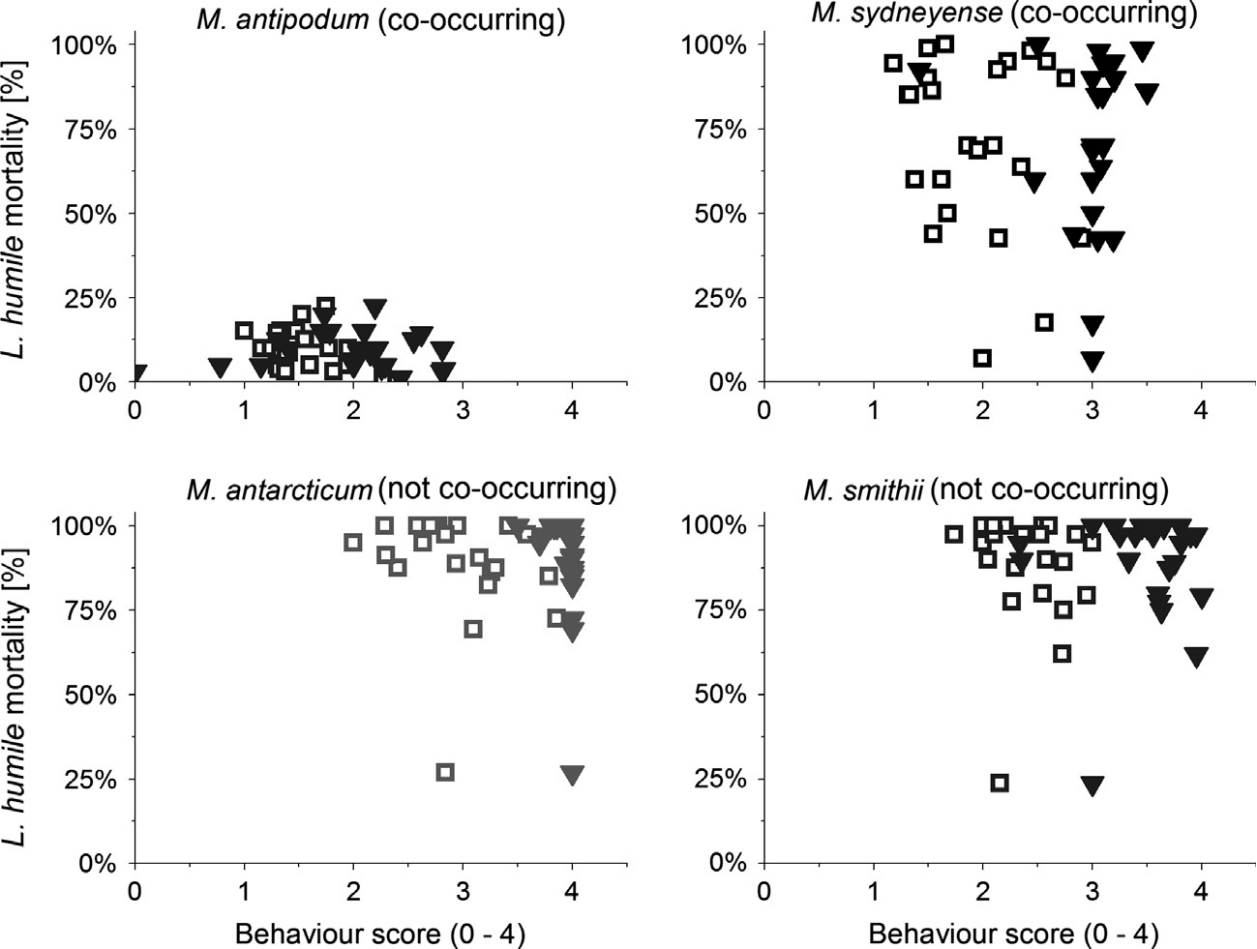
Argentine ant mortality in arena fights. Mortality of Argentine ant workers in arena fights against *Monomorium* species depending on average individual aggression within the same replicate (open squares) and average maximum aggression within the same replicate (closed triangles). Behavior scores were taken as follows: 0 = no interest or aggression; 1 = interest shown via antennation; 2 = ant retreats quickly; 3 = lunging, biting or leg pulling, raising of gaster, and exuding venom; and 4 = prolonged (>5 sec) incidences of aggression, individuals locked together, and fighting.

For Argentine ants, different patterns of mortality and behaviors for encounters with all four species were also observed (Fig.4). Worker mortality varied significantly depending on the *Monomorium* species encountered (generalized linear model, *N* = 96, df = 3, Wald chi-square = 170.54, *P* < 0.001). We observed the lowest mortality in Argentine ants when interacting with *M. antipodum* (0.9%) and the highest mortality when encountering *M. smithii* (89%). We only found an effect of individual aggression and Argentine ant density on Argentine ant mortality when facing *M. antarcticum* (generalized linear model, *N* = 24, df = 1, Wald chi-square = 3.44, *P* = 0.063; covariate Argentine ant density df = 3, Wald chi-square = 8.53, *P* = 0.036).

## Discussion

Our study showed differences in the behavior of the two *Monomorium* species co-occurring with the invasive Argentine ant. The co-occurring species utilize much more potent venom, and the aggression display between their workers and Argentine ants is lower than that of the two species which do not co-occur. A summary of our findings is presented in Table3. Our first hypothesis was that species with higher venom toxicity would have a higher survival rate when engaging Argentine ants. We based this initial hypothesis on the assumption that the two *Monomorium* species (*M. antarcticum* and *M. smithii*) displaying negative co-occurrence with Argentine ants might do so because *Monomorium* ants are able to exclude Argentine ants from their habitat through biotic resistance, by means of venom application. Our analysis of venom survival experiments showed that the venom of the two *Monomorium* species, which do not co-occur (*M. antarcticum* and *M. smithii*), is less toxic to Argentine ants than the venom of the two *Monomorium* species that do co-occur (*M. antipodum* and *M. sydneyense*). There is an alternative explanation for the venom toxicity/spatial patterns, which may also explain our results. Namely, it is possible that *Monomorium* species that display negative co-occurrence with Argentine ants do so, because they are poorly defended and are therefore excluded by the Argentine ant. Indeed, there are numerous examples of the Argentine ant competitively excluding other ant community species (Holway et al. 2002a). Species who have been reported to resist such exclusion, such as the winter ant (*Prenolepis imparis*), utilized high venom toxicity (Sorrells et al. 2011). However, if the observed co-occurrence/exclusion patterns were primarily governed by venom toxicity, as we had initially hypothesized, we would have expected different mortality patterns for the two *Monomorium* species with lower venom toxicity (Fig.2). Specifically, if Argentine ants excluded species with low venom toxicity, we would expect their survival to be much higher when interacting with *M. antarcticum* and *M. smithii* and as a corollary of this, would expect those species to have a higher mortality relative to the species which are better defended through their toxicity. This does not appear to be the case in our model, which indicates that other factors, such as aggression, are more influential in the outcome of interactions. Indeed, the counterintuitive significant negative correlation between *Monomorium* venom toxicity and Argentine ant survival can very likely be explained by the differences in aggressiveness between those *Monomorium* species when interacting with Argentine ants. Comparing our results with studies which previously investigated physiological functionality of ant venoms (Lind 1982; Lebrun and Cattaert 1997) further supports our findings, as it is highly plausible that the increased chain lengths of *M. antipodum* and *M. sydneyense* pyrrolidines (**6**, **7**, **8**, and **9**; Fig.1) should result in increased toxicity. The differences we found in the venom composition of *M. smithii* compared to a previous study (Jones et al. 1990) are likely due to the possibility that there are two cryptic species, which are morphologically similar and given the current taxonomic keys, can only be identified to *M. smithii*. While it is a possibility that species with less toxic venom might simply deliver more of it, this was not tested in this study. However, even if this were the case, a species delivering more of less effective venom would consequently need more extensive venom reserves to mitigate the risk of exhausting them too quickly. As all tested ants were of relatively similar size, we would expect them to have comparable venom reserves. The result that increased toxicity did not translate into an increase in the survival of *Monomorium* and higher *Monomorium* toxicity was even correlated with higher Argentine ant survival suggests that venom toxicity is not the main factor driving the co-occurrence patterns between *Monomorium* and Argentine ants. However, toxicity may nonetheless play a still play a more limited role in mediating the outcome of aggressive interactions as has been shown by other studies (Andersen et al. 1991; Andersen and Patel 1994). Given this result, other factors, such as aggressive behavior, must be taken into account to explain the interactions between these species and obtain a better understanding of how venom chemistry might influence community assembly.

**Table 3:** Summary of experimental results and proposed interpretation

Species	Observed spatial patterns	Venom toxicity	Venom utilization	Arena trials			Proposed interpretation
				Observed aggression	Mortality of Argentine ants	Mortality of *Monomorium*	
*Monomorium antarcticum*	Does not co-occur	LOW (LD50 8.14 μg/μL)	Stinging, No change	High	High	Low	*M. antarcticum* excludes Argentine ants
*Monomorium smithii*	Does not co-occur	LOW (LD50 9.76 μg/μL)	Gaster flagging/Stinging	High	High	High	Mutual exclusion possible
*Monomorium antipodum*	Co-occurs	HIGH (LD50 2.95 μg/μL)	No venom/Gaster flagging, Gaster flagging increases	Low	Low	Low	Coexistence
*Monomorium sydneyense*	Co-occurs	HIGH (LD50 2.89 μg/μL)	Stinging, No change	Medium	Medium	Medium	Coexistence

Columns display the four *Monomorium* species occurring in New Zealand, the observed co-occurrence patterns with the invasive Argentine ant, relative toxicity and LD50 of *Monomorium* venom against Argentine ants found in venom toxicity tests, most common means of venom application by *Monomorium* observed in arena trials as well as observed changes of behavior with increased Argentine ant abundance, aggression levels in arena trials, Argentine ant mortality in arena trials, *Monomorium* mortality in arena trials, and proposed interpretation of the combined factors in regard to the observed spatial patterns. The given classifications of “high,” “medium,” and “low” are given as relative approximations to the average observed toxicity/aggression/mortality for the respective species.

We hypothesized that the likelihood of *Monomorium* species surviving aggressive interactions with Argentine ants would differ depending on venom utilization. While some animals primarily use their venom in close, physical interactions, some also have the ability to shoot or spit their venom over a larger distance (Warrell 2007). The way venom is utilized in different circumstances, or by certain species, might be an important aspect of venom efficacy, which was previously poorly investigated. Previous studies on the most effective way to use venom have primarily focused on venom optimization from a cost-benefit approach (Wigger et al. 2002; Longson and Joss 2006; Morgenstern and King 2013). However, not only the metabolic costs of venom production need to be considered, but also the cost benefit of the utilization. For example, two *Monomorium* species which have not been observed to co-occur with Argentine ants, *M. antarcticum* and *M. smithii,* both used their venom primarily in close encounters to sting Argentine ant workers while grappling them. Although the toxicity of their venom appeared to be lower in our trials, the direct injection into their opponents’ body bypasses the bodies’ outer barrier and seldom misses its target. On the other hand, using venom as a distance weapon bears less risk of injury if successful. There are many inst ances of spray or squirt weapons in animals, although not all are venomous (Aneshansley et al. 1969; Sherbrooke and Middendorf 2004; Westhoff et al. 2005; Suter and Stratton 2009).

It has previously been shown that Argentine ants can subdue larger species in aggressive interactions through numerical superiority (De Kock 1990). We therefore expected that, if venom does not provide a significant advantage, aggressive interactions should result in a lower survival rate of the species engaging Argentine ants, especially at higher densities. Contrarily, our results showed that only in two of four *Monomorium* species did the increase in aggressive interactions actually result in an increase in *Monomorium* worker mortality. However, increased aggression also caused a great increase in mortality for Argentine ant workers when engaging *M. antarcticum*. Several nonmutually exclusive explanations exist for these results. For example, it is possible that other factors such as size or physical prowess play an important role, and Argentine ants are consequently only capable of dominating other ant species when occurring in overwhelming abundance (Walters and Mackay 2005). Alternatively, it is possible that not all species elicit the same strong aggressive response from Argentine ants. In a previous experiment, we showed that Argentine ants react differently to competitors in field experiments and may chose to ignore or avoid some species if met under natural conditions (Westermann et al. 2014).

The combination of the factors we investigated (venom toxicity, venom utilization, aggression, and survival during interactions) allows for a different explanation underlying the observed distribution patterns between *Monomorium* and Argentine ants (Table3). For instance, in the case of *M. antarcticum*, the relatively low venom toxicity makes chemical interactions an unlikely explanation for biotic resistance. However, our arena tests showed that *M. antarcticum* can successfully defend itself against Argentine ants, even when vastly outnumbered. This success may well lead to biotic resistance, but on the basis of physical aggression. For *M. smithii*, the pattern is unclear, as high aggression and mortality on both sides could lead to either side excluding the other. However, given the displacement of other species by Argentine ants (Holway 1999; Holway et al. 2002a; Rowles and O’Dowd 2007), it appears likely that in this case, the numerical superiority of the Argentine ant would lead to the exclusion of *M. smithii*. In *M. antipodum,* the low aggression/mortality on both sides (Table3) suggests little interference competition with the Argentine ant; therefore, co-occurrence can be explained if niche/resource partitioning were to be assumed. For *M. sydneyense*, the higher venom toxicity against Argentine ants may give workers an advantage when harassed, which allows *M. sydneyense* to co-occur with the Argentine ant.

Many different factors contribute to the success of an invasive species. Similarly, the ability of some species to co-occur and offer resistance against an invasive species is multifaceted. Previous studies have shown that venom plays an important role in ant community composition (Andersen and Patel 1994; Holway 1999; Sorrells et al. 2011). The underlying mechanisms for the co-occurrence patterns cannot, however, be explained by venom toxicity alone, although there is some evidence in *M. antipodum* that venom behavior might influence survival chances.

Other studies have shown that the way species use their venom influences their competitive chances and determines community structure. For example, the tawny crazy ant (*Nylanderia fulva*) has been found to be able to detoxify the venom of its fiercest competitor, the red imported fire ant (*Solenopsis invicta*), by covering itself with its own venom (LeBrun et al. 2014). Aggressive interactions also contribute partially to the co-occurrence patterns. However, the overall success in engagements is likely determined by a multitude of intertwined factors such as size and physical prowess, numerical abundance (Sagata and Lester 2009), behavior, defensive strategies (Barbieri et al. 2013), and toxicity, which are difficult to assess individually. Our study showed that different factors and strategies contribute to the ability of interacting organisms to withstand the pressure of a dominant invader at high abundance. While toxicity is one factor, other strategies such as avoidance might also allow co-occurrence, while strong aggressive interactions likely result in territorial exclusion.
